# Psychometric Evaluation of the Team Member Perspectives of Person-Centered Care (TM-PCC) Survey for Long-Term Care Homes

**DOI:** 10.3390/healthcare6020059

**Published:** 2018-06-06

**Authors:** Veronique M. Boscart, Meaghan Davey, Jenny Ploeg, George Heckman, Sherry Dupuis, Linda Sheiban, Jessica Luh Kim, Paul Brown, Souraya Sidani

**Affiliations:** 1 Schlegel Centre for Advancing Seniors Care, 299 Doon Valley Drive, Kitchener, ON N2G 4M4, Canada; zehrm1@mcmaster.ca (M.D.); lsheiban@conestogac.on.ca (L.S.); 2 Schlegel-University of Waterloo Research Institute for Aging, 250 Laurelwood Drive, Waterloo, ON N2J 0E2, Canada; ggheckma@uwaterloo.ca; 3 Schlegel Villages, 325 Max Becker Dr, Kitchener, ON N2E 4H5, Canada; Jessica.luhkim@schlegelvillages.com (J.L.K.); paul.brown@schlegelvillages.com (P.B.); 4 School of Nursing, McMaster University, 1280 Main St. W., Hamilton, ON L8S 4K1, Canada; ploegj@mcmaster.ca; 5 Faculty of Applied Health Sciences, University of Waterloo, 200 University Avenue West, Waterloo, ON N2L3G1, Canada; sldupuis@uwaterloo.ca; 6 Daphne Cockwell School of Nursing, Ryerson University350 Victoria St, Toronto, ON M5B 2K3, Canada; ssidani@ryerson.ca

**Keywords:** psychometric properties, long-term care homes, person-centered care, long-term care, care quality, measurement

## Abstract

Person-centered care (PCC) is fundamental for providing high-quality care in long-term care homes. This study aimed to evaluate the psychometric properties of an 11-item Team Member Perspectives of Person-Centered Care (TM-PCC) survey, adapted from White and colleagues (2008). In a cross-sectional study, 461 staff from four long-term care homes in Ontario, Canada, completed the TM-PCC. Construct validity and internal consistency of the TM-PCC were examined with a principal component analysis and Cronbach’s alpha coefficient. Findings revealed a three-component structure with factor 1, Supporting Social Relationships; factor 2, Familiarity with Residents’ Preferences; and factor 3, Meaningful Resident–Staff Relationships. The TM-PCC, as compared to the original survey, presented with less components (i.e., did not address Resident Autonomy, Personhood, Comfort, Work with Residents, Personal Environment, and Management Structure), yet included one new component (Meaningful Resident–Staff Relationships). The TM-PCC has a similar internal consistency (Cronbach’s alpha coefficient 0.82 vs. White et al. 0.74–0.91). The TM-PCC can be used to assess PCC from the staff’s perspective in long-term care homes.

## 1. Introduction

Long-term care homes (also referred to as nursing homes) provide medical care and activities of daily living (ADL) support to over 1.4 million Americans and more than 224,000 Canadians [1,2]. Many of these seniors are frail and have complex medical conditions, and therefore depend on staff assistance [1,3]. Person-centered care (PCC) has been recognized as a fundamental element in providing high-quality care tailored to residents’ needs [4,5]. PCC is generally defined as care which is responsive to the person’s well-being, which includes meaningful social interactions, shared choice and decision-making, and accommodating a person’s preferences, values and beliefs [4,5]. PCC moves beyond a biomedical approach to include the person’s autonomy and ensure that their overall well-being is being cared for [5]. One literature review by Kogan and colleagues [5] describes 15 definitions of PCC, all encompassing at least one of 17 different principles or values that are central to PCC. Some of these principles include “whole-person care”, “respect and value”, “dignity” and “self-determination”.

PCC has been described as challenging to implement in home and community care settings due to barriers such as regulation of services [6]. However, PCC has been successfully implemented in long-term care settings where older adults with complex care needs receive multidisciplinary services [5]. In 2007, 31% of long-term care homes in the United States self-reported as having adopted a PCC or ‘resident’-centered care (RCC) approach, although there is little consistency in the use of terms or the implementation of the PCC principles [7]. Although similar statistics are lacking for Canadian long-term care homes who have adopted these practices, PCC is widely advocated to improve care quality [8].

Several studies have described the benefits of PCC implementation in long-term care homes: improved residents’ quality of life and family satisfaction, reduced levels of boredom and helplessness for residents, and improved staff sense of empowerment and job satisfaction [4,5,9,10,11]. Yet, methodologically, little evidence is available on the initiatives to integrating PCC into practice, making it difficult to evaluate any fidelity and effectiveness [4,5].

In addition, few valid and reliable assessment tools are available to evaluate the implementation of PCC practices in long-term care homes [12]. A plethora of PCC assessment tools across health care disciplines aim to evaluate different components of PCC [13]. However, most tools are designed for acute care settings, and of those designed for long-term care homes, few have shown validity and reliability [13]. Long-term care home populations, staffing and work context are vastly different from acute care environments. Staff provide comprehensive and complex care to a large number of residents and their families throughout extended time periods; staff-turnover is often high, and most care is provided by certified nursing assistants with limited formal education [14].

A literature search revealed additional assessment tools specific to long-term care/nursing homes (Table 1) [15,16,17,18,19,20,21,22,23,24,25,26]. Despite the availability of these instruments, some overall concerns must be noted. The instruments vary in the PCC dimensions measured, number of items, the intended user (i.e., resident, staff, facility), method of administration (i.e., interview, observation, questionnaire), and are designed for residents with different diagnoses (i.e., dementia, communication disorders). For example, the number of dimensions varies from measuring one very detailed construct (i.e., person-centered communication) to eight different constructs [23,25,27]. Furthermore, most tools appear to be used in isolation, suggesting that researchers prefer to use their own tool to address outcomes specific to the research question under investigation. The performance of the tools varied widely as well; the psychometric properties of three (18.8% of the 16 tools) were not evaluated. Of those tools, which were evaluated, Cronbach’s α ranged from 0.62 to 0.94. Additionally, there does not appear to be a clear rationale for the choice of one tool over another, besides the user’s preference.

Two of the more commonly cited tools include the Person-Directed Care Measure and the Person-Centered Care Assessment tool [27,28]. The Person-Directed Care Measure was designed in the United States for completion by staff and includes 64 items on resident autonomy, personhood, knowing the person, comfort, supporting relationships, personal environment for residents, work with residents, and management structure [27]. This measure has a high internal consistency reliability (Cronbach’s α 0.74–0.91) and the factors explained moderately high variance (61%) in the items’ responses [27]. Sullivan and colleagues (2012) tested the Person-Directed Care Measure in Veterans Health Administration long-term care homes and strongly recommended the deletion of some conceptually redundant items and afterwards assessing its construct validity; although, they did not specify which items to remove or an ideal number of items.

The second tool, the Person-Centered Care Assessment tool, was designed in Europe for completion by staff and includes 13 items on personalized care, and organizational and environmental support [28]. This tool has a high internal consistency reliability (Cronbach’s α 0.83) and validity [28]. The original version (developed and tested in Australia) had a limited response rate of 21% in the first validation study and one of the subscales did not reach the Cronbach’s alpha cut-off criterion of 0.7. This version was subsequently adjusted for use in Europe, and upon testing, had a high response rate (88%) and some evidence of reliability and validity [28].

In summary, while there are many PCC assessment tools available, few (<20) are validated and specific to long-term care homes. Because of the complex nature of long-term care in Canada, we deemed it necessary to develop a context-specific PCC tool. The Person-Directed Care Measure [27] has a high internal consistency and reliability, indicating redundancy amongst its items. The Person-Centered Care Assessment tool [28] is limited in its use because of under-representation in North American studies. A parsimonious, validated assessment tool specific to Canadian long-term care homes would aid in the feasible measurement and evaluation of PCC practices as perceived by staff.

We adapted the Person-Directed Care Measure [27] to develop the ‘Team Member Perspectives of Person-Centered Care’ (TM-PCC) survey, a brief and validated tool to assess and evaluate dimensions of PCC. The choice to base the TM-PCC on the Person-Directed Care Measure [27] was built on the fact that this tool is commonly used, yet needed to be shortened by removing redundant items [12]. There are several advantages to administering a short survey to long-term care home staff: increased likeliness of completion, respondents are more likely to be engaged throughout the survey, and less competition for staff’s already limited availability. Additionally, the Person-Directed Care Measure [27] covers a wide range of PCC practices and has shown strong psychometric properties. Therefore, the primary objectives of this study were to adapt the Person-Directed Care Measure [27] to develop the TM-PCC survey and test the TM-PCC survey’s psychometric properties with a group of long-term care home staff. Specifically, we aimed to evaluate internal consistency and construct validity. The study was conducted in accordance with the Declaration of Helsinki, and the protocol was approved by the Tri-Council Ethics Board at the agency (REB-118).

## 2. Materials and Methods

### 2.1. Tool Development

The original 64-item Person-Directed Care Measure [27] includes eight PCC dimensions. The measure was designed for staff to rate their own care practices. Each item was rated on a Likert-type scale as 1 = ’Never’, 2 = ’Rarely’, 3 = ’Sometimes’, 4 = ’Almost all the time’, 5 = ’All the time’.

An expert panel consisting of long-term care home staff, residents and families, administrators and researchers selected a subset of 11 items from the Person-Directed Care Measure [27]. The 11 items were selected based on the perceived importance of which the item specifically addressed PCC practices, validation of the item’s importance in the literature, and on the individual item’s psychometric performance. A key resource was the work of Bangerter and colleagues (2015), which surveyed long-term care home residents to identify their preferences with respect to care interactions and which emphasized the importance of care partners knowing the residents’ preferences [29]. In addition, the expert panel and the literature review supported the decision to create three new items to capture the relationships between staff and residents; an important aspect of PCC not fully addressed in the Person-Directed Care Measure [27]. Specifically, White and colleagues (2008) addressed supportive relationships, which focused on friends and family, but did not include the relationships between residents and staff in their Person-Directed Care Measure [27]. Other tools have incorporated items on the staff–resident relationship [20,22,23], further strengthening the importance for inclusion. Table 2 provides an overview of supporting references for the items [21,29,30,31,32,33,34,35].

The final TM-PCC survey included a total of 14 items exploring PCC practices from the perspective of the staff. Each item was rated similarly to the original survey on a Likert-type scale as 1 = ’Never’, 2 = ’Rarely’, 3 = ’Sometimes’, 4 = ’Almost all the time’, 5 = ’All the time’. Estimated completion time was five to ten minutes. The TM-PCC survey is designed to be completed by care team members, specifically registered nurses (RNs), licensed practical nurses (LPNs) and certified nursing assistants (CNAs). Although CNAs provide the majority of residents’ care, all care members were included in the sample, as they collaborate as a team. RNs are typically involved when residents’ care needs are not well-defined, when health conditions are not well-controlled or require frequent monitoring, and when outcomes are unpredictable [36]. LPNs support residents with well-defined care needs with little fluctuation in conditions and when there is a low risk of negative outcomes [36]. CNAs provide assistance with residents’ activities of daily living and provide the most direct care time [14].

The readability of items was assessed using Flesch reading easy score, which is widely established as an accurate measure [37]. Readability was rated as ‘standard’ by the Flesch reading easy score suggesting that the content of the items was understandable at an 8th to 9th grade level [37].

### 2.2. Sampling Strategy

The staff sample was randomly selected from 4 long-term care homes in the province of Ontario, Canada (long-term care homes were assigned numbers and units were assigned letters). Eight of the fifteen surveyed units were dementia care units. Research assistants approached 750 care staff (RNs, LPNs and CNAs) to obtain written consent and invited them to conduct the TM-PCC survey. Data were collected from 1 July 2014 to 30 June 2015. As this was part of a larger study, more details on the study methods and sampling are described elsewhere [38].

### 2.3. Analytical Strategy

Only complete data were included in the analysis. Of the invited care staff (n = 750), 461 completed a TM-PCC (response rate 61.5%). Eighty-two (17.8%) surveys were not usable due to missing information. Of the remaining surveys, 379 (82.2%), descriptive statistics were calculated for respondents’ demographics and for each item on the TM-PCC, as well as the subscales reflecting PCC dimensions. Principal component analysis was chosen a priori to evaluate construct validity because it is a variable reduction technique for large samples when variables are highly correlated; it generates component scores which are a linear combination of the observed variables [39]. Unlike factor analysis, it does not assume underlying latent constructs in the data [39]. Factors were extracted based on the Kaiser criterion (eigenvalues of factors >1), inspection of the scree plot, and percentage of variance explained (>10%) [40]. Items were assigned by component loadings of 0.40 or greater and deleted if they loaded on more than one component where the difference was <0.20 [40]. Results are presented with orthogonal rotation for interpretability and to preserve variable communalities [41]. Internal consistency was examined using a Cronbach’s alpha coefficient. All data analysis was performed using SAS 9.4 software (SAS Institute, Cary, NC, USA).

## 3. Results

### 3.1. TM-PCC Survey Results

A total of 750 staff were invited to participate in the surveys. Several participants (289, 38.5%) did not consent, leaving a total of 461 survey responses: 33 (7.1%) RNs, 79 (17.1%) LPNs and 349 (75.7%) CNAs. The majority of participants were employed on a full-time basis (83.1%). The ratio of full-time and part-time did not appear to vary by long-term care home. With respect to years of participant experience working in long-term care homes, the number of staff with over ten years of experience ranged from 5.6% to 32.4% across long-term care homes. A significant proportion of participants had no more than three years of experience in the current long-term care home (26.0–53.9%). Generally, most staff were female (89.8–94.7%). Most staff appeared to be between 25 and 45 years of age (35.5–84.0%). 

Areas of PCC which all staff rated highest included item 4 (i.e., “I quickly help __ of my residents to the toilet when they request or need help”), with a score of 4.2–4.4 out of five across four long-term care homes (Table 3). Item 12 (i.e., “I look after the same residents from day to day”) was rated 4.1–4.3 out of five. Item 15 (i.e., “I am able to build fulfilling relationships with residents”) was rated 4.0–4.3 out of five. Lastly, item 16 (i.e., “I can learn from residents and their family members and incorporate this caring into my daily routine”) was rated 4.0–4.2 out of five. Item 8 rated the lowest (i.e., “I help __ of my residents stay connected to previous associations”) with a rate of 2.2–2.8 out of five. All TM-PCC survey scores are described in Table 3.

### 3.2. Component Structure

In the first principal component analysis, three items (item 5: “I know when __ of my residents need to use the toilet, even if they cannot speak”, item 6: “I can calm __ of my residents if they feel agitated or upset”, and item 11: “I spend time talking or just being with __ of my residents”) each loaded on two components. The difference in the loading was <0.2, suggesting that these three items could be potentially removed. Although these items are conceptually important, when removing these items the eigenvalues and inspection of the scree plot suggested a three-component structure for the TM-PCC survey. The component pattern matrix is displayed in Table 4. The cumulative variance explained by the three components was 61.5%.

Four items loaded on the first component, contributing 36.5% to the total explained variance, and with an eigenvalue of 4.0. The loadings of four items, that is, loadings of the item 5: “I help my residents stay connected to their families”, item 6: “I help my residents stay connected to previous associations”, item 7: “I help my residents keep family members as part of their life”, and item 8: “I help my residents spend time with people they like”, ranged from 0.66 to 0.86. As a result, this component was named *Supporting Social Relationships*.

There were four items which loaded on the second component. This component contributed 13.9% to the total explained variance and the eigenvalue was 1.5. Loadings for item 1: ‘I know the preferred habits for my residents’, item 2: ‘I know my residents’ favorite foods’, item 3: ‘I know my residents’ favorite music’, and item 4: ‘I quickly help my residents to the toilet when they request or need help’, ranged from 0.55 to 0.81. As a result, this component was named *Familiarity with Residents’ Preferences*.

There were three items which loaded on the third component. This component contributed 11.1% to the total explained variance and the eigenvalue was 1.2. Component loadings of item 9: ‘I look after the same residents from day to day’, item 10: ‘I am able to build fulfilling relationships with residents’, and item 11: ‘I can learn from residents and their family members and incorporate this caring into my daily routine’, ranged from 0.63 to 0.84. As a result, this component was named *Meaningful Resident–Staff Relationships*.

### 3.3. Internal Consistency Reliability

Communality estimates for each item ranged from 0.41 to 0.77. Cronbach’s alpha coefficient for all 11 items was 0.82. Internal consistency for the three components was generally high and ranged from 0.62 to 0.83. Average scores in each construct were high and ranged from 3.56 to 4.17 out of five. The descriptive statistics and Cronbach’s alpha coefficient for each of the components are described in Table 5.

## 4. Discussion

Given the importance of PCC practices to enhance care quality for residents in long-term care homes, few validated tools are developed specifically to measure PCC in these settings. The need to develop a brief, valid and reliable tool to measure a wide range of PCC practices led to the development of the TM-PCC survey. The development was based on theoretical and evidence-based items, founded in the Person-Directed Care Measure [27], as well as a literature review, and decided by an expert panel to be specific to PCC in long-term care homes. The goal of this study was to develop and validate a tool to measure PCC practices to be able to assess implementation and evaluation of PCC interventions. TM-PCC survey data were collected from 461 staff in four long-term care homes to test the survey for its psychometric properties.

In terms of respondents, there was high variability across units and homes in terms of years of experience. Other staff characteristics such as age, gender and shift type did not appear to vary much across units or homes, with a predominantly female staff employed on full-time shifts. A lack of research on the variability of long-term care home staff prevents larger comparisons. However, these demographics findings reflect what is generally reported in practice and suggest that the results are generalizable to other long-term care homes.

The analysis provided strong evidence to support the internal consistency and construct validity of the TM-PCC survey. A principal component analysis revealed a three-factor structure, with the cumulative variance explaining 61.5%. The internal consistency was high with a Cronbach’s alpha of 0.82. These findings compare favorably with the Person-Directed Care Measure [27], which demonstrated a Cronbach’s alpha coefficient of 0.74–0.91 and variance explained of 61%.

For the TM-PCC, the first component, *Supporting Social Relationships* presented with high loading from four items related to helping residents stay connected to friends and family. It explained 36.5% of the total variance. Residents value meaningful relationships and are dissatisfied when there are no such opportunities [42]. Similar to the TM-PCC survey, Sidani and colleagues (2014) included social relationships as part of a larger holistic care construct. Additionally, social environment and individual aspects of the person were determined as important principles central to PCC [5]. Maintaining social relationships is a recognized aspect of care models, building on relational and humanistic theories [42,43].

The second TM-PCC survey component, *Familiarity with Residents’ Preferences* presented with a high loading on four items related to residents’ preferred habits, food and music. This component explained 13.9% of the total variance. Knowing and accommodating residents’ preferences is central to the idea of PCC [5,13]. For example, Kogan and colleagues (2015) describe how respect and value is a principle central to PCC. Rokstad and colleagues (2012), Van Haitsma and colleagues (2014), and Yeung and colleagues (2016) included items in their surveys to support residents’ preferences, while Sidani and colleagues (2014) operationalized this as an element of ‘responsive care’ in their scale. Palmer and colleagues (2017) extended the concept further to evaluate the communication surrounding the delivery of care based on resident preferences. Additional items on how staff deliver customized care knowing resident preferences as well as items on staff’s knowledge of residents’ beliefs and values could add depth and validity to this construct.

The third component, *Meaningful Resident–Staff Relationships,* had high loadings from three items related to consistent care assignment, learning from residents and building relationships with residents. It contributed 11.1% to the total explained variance. In a systematic review by Kogan and colleagues [5], facilitating enriched relationships was an important value within the definition of PCC. Emotional caring and mutual sharing of personal information is central to residents feeling satisfied with their care and well-being [42]. De Brouwer and colleagues (2017) included constructs on relationships in their scale. The nature of the relationship between staff and residents is a determinant of care delivery: understanding the resident as a person engages staff to discover residents’ preferences and provide responsive care [42]. Developing meaningful relationships depends on staff having sufficient time to engage with residents, as well as requires consistent care assignments, relating to the construct on organizational support [42].

A few limitations need to be noted for this study. Although the TM-PCC survey did not include items related to resident autonomy, shared decision-making, or resident engagement in activities, it did emphasize elements of PCC such as meaningful social interactions and recognizing residents’ preferences [4,5]. Another limitation of the study relates to the absence of test–retest validity testing. When compared with other tools designed by researchers, the TM-PCC survey performs moderately well. The internal consistency and percent variance explained were high when compared to other tools [15,16,19,21,22,26]. Additionally, the TM-PCC employed Likert-type scales which are simple to construct and easy to complete for staff; however, this style of questioning can be subject to central tendency bias (i.e., where participants avoid extreme response categories) which could make it difficult to monitor improvement in scores over time. As well, the choice to perform a principal component analysis allowed for reducing the number of observed variables into a smaller number that accounted for most of the variance in the dataset and made no assumptions concerning an underlying causal structure. However, this strategy required a large sample size (i.e., ideally five times the number of survey items) and dropped some items, which is why we included multiple items for each construct we hoped to measure [39]. 

Additionally, there was a significant number of staff who chose not to participate in the study which could have biased results. Of the participants who did not consent, ten staff indicated some demographic information; however, this is not a large enough sample to explain why some staff chose not to consent. Because completing research surveys requires time away from residents, it is unclear if staff who chose not to consent were less motivated to provide person-centered care than staff who completed surveys. This further emphasizes the need for surveys to be concise in order to maximize completion rates and ease the burden on care staff. As well, the TM-PCC evaluated staff perspectives only. Future tools could expand upon resident and family perspectives.

We also found that staff scores were generally high, suggesting a potential social desirability bias. One could emphasize to staff that the survey’s purpose is to identify areas where one can improve rather than judging an individual’s performance. This likely had minimal effects on the validity of the tool since decreased variability in scores would decrease the risk of a type 1 error. As the survey was only administered to four long-term care homes in one Canadian province, results may vary among countries, long-term care home models, more or less experienced staff, different staffing ratios, or variations in PCC operationalization. In this study, we did not focus on non-nursing staff responses (i.e., other staff on the team such as kinesiologists, housekeeping, physicians, etc.). Finally, perceptions from the participants about the acceptability of completing the measure were not sought. However, this is valuable information and will be looked at in further research for the future utility of TM-PCC.

In summary, the TM-PCC survey is a brief, validated tool to assess PCC practices in long-term care homes from a staff’s perspective. The survey includes similar constructs as other tools and has a high internal consistency and construct validity. The three components in the adapted TM-PCC survey mirror key principles of PCC that were described in a literature review completed by Kogan and colleagues (2015). It can be used in long-term care homes interested in monitoring or implementing PCC interventions. Using validated tools such as the TM-PCC survey promotes accurate representations of the PCC delivered in the care settings as well as staff’s perceptions towards PCC. The results of this study can help identify staff, teams and leadership in long-term care homes to identify practices where teams are confident in PCC, as well as those where supports may be needed.

## 5. Conclusions

The TM-PCC survey was considered a psychometrically valid tool for use in long-term care homes. The use of valid, standardized tools allows for the comparison of care between different care settings as well as the comparison of changes in care practices within the same setting.

## Figures and Tables

**Table 1:** Person-Centered Care Tools for Use in Long-Term Care/Nursing Homes.

Author, Year	Name	Country of Origin	No. of Items	User	Constructs	Performance
De Witte et al., 2006	Client-Centered Care Questionnaire	The Netherlands	15	Client	Decision-making, communication	Cronbach’s α 0.94, variance explained 58%
White et al., 2008	Person-Directed Care Measure	USA	64	Staff Facility	Personhood, knowing the person, comfort care, autonomy, supporting relationships, staff work with residents, personal environment for residents, management/structure	Cronbach’s α 0.74–0.91, variance explained 61%
Bradford Dementia Group, 1997	Dementia Care Mapping	England	63	Staff	Mood enhancers, behaviors, personal detractions and enhancers	Intraclass correlation coefficient 0.70
Rokstad et al., 2012	Person-Centered Care Assessment Tool	Norway	13	Staff	Personalized care, organizational and environmental support	Cronbach’s α 0.83, variance explained 45%
Bergland et al., 2012	Person-Centered Climate Questionnaire-Staff Version	Norway	14	Staff	Climate of safety, climate of everydayness, climate of community	Cronbach’s α 0.92, Spearman’s correlation 0.76, variance explained 68%
Bergland et al., 2014	Person-Centered Climate Questionnaire-Patient Version	Norway	17	Staff	Climate of safety, climate of everydayness, climate of hospitality	Cronbach’s α 0.84, item-total correlation 0.10–0.68
Hwang et al., 2012	Elderly Resident-Perceived Caring Scale	Taiwan	14	Staff	Comforting, encouraging	Cronbach’s α 0.92, variance explained 64.3%
Kurokawa et al., 2013	Personhood Questionnaire	Japan	17	Staff	Habit, lifestyle, interest, character style	Cronbach’s α 0.89
Gaugler et al., 2013	CARES^®^ Observational Tool	USA	16	Staff	Compassionate encounter	Intraclass coefficient 0.77
Van Haitsma et al., 2014	America’s Nursing Homes, PCC toolkit	USA	16	Staff	Residents’ preferences	None
Yeung et al., 2016	Eden Warmth Survey-Residents	New Zealand	22	Client	Satisfaction with staff, care, medical attention, support, activities, meals	Variance explained 57.9%
De Brouwer et al., 2017	Essentials of Magnetism II	The Netherlands	58	Staff	Clinically competent peers, collaborative nurse–physician relationships, clinical autonomy, nurse manager support, control over nursing practice, perceived adequacy of staffing, support for education, patient-centered culture	Cronbach’s α 0.92
Palmer et al., 2017	Supporting Choice Observational Tool	USA	9	Client	Formative assessment of aspects of daily life, staff offering a choice, resident accepting a choice, staff enabling the choice	None
Skinder-Meredith et al., 2007	Patient-Centered Communication	USA	Unknown	Staff	Tools and strategies used to facilitate communication	None
Sidani et al., 2014	Unnamed	Canada	27	Staff	Holistic care, collaboration, responsive care	Variance explained for each item 37.6%, 27.3%, 37.5%
Miller et al., 2014	Unnamed	USA	Unknown	Facility	Environment, staff empowerment	Cronbach’s α 0.62

**Table 2:** Literature Supporting Team Member Perspectives of Person-Centered Care Survey Items.

Survey Items	Supporting References
I know the preferred habits for __ my residents	Bangerter et al., 2015
I know __ of my residents’ favorite foods	Bangerter et al., 2015; Hung et al., 2016
I know ___ of my residents’ favorite music	Bangerter et al., 2015; Van Haitsma et al., 2014
I quickly help __ of my residents to the toilet when they request or need help	Bangerter et al., 2015; Nakrem et al., 2011
I know when __ of my residents need to use the toilet, even if they cannot speak	Bangerter et al., 2015
I can calm __ of my residents if they feel agitated or upset	Bangerter et al., 2015
I help __ of my residents stay connected to their families	Nakrem et al., 2011
I help __ of my residents stay connected to previous associations	Nakrem et al., 2011
I help __ of my residents keep family members as part of their life	Nakrem et al., 2011
I help __ of my residents spend time with people they like	Nakrem et al., 2011
I spend time talking or just being with __ of my residents	Edvardsson et al., 2016; Nakrem et al., 2011
I look after the same residents from day to day	Hung et al., 2016; Van Haitsma et al., 2014
I am able to build fulfilling relationships with residents	Donnelly et al., 2016; Simmons et al., 2014; Yoon et al., 2015
I can learn from residents and their family members and incorporate this caring into my daily routine	Simmons et al., 2005; Edvardsson et al., 2016

**Table 3:** Description of TM-PCC Survey Results.

	Long-Term Care Home (n, %/SD)				Missing Values (n)
	1	2	3	4	
	(n = 179)	(n = 123)	(n = 38)	(n = 121)	
**Profession**					0
Registered Nurses	16 (8.9)	6 (4.9)	5 (13.2)	6 (5.0)	
Licensed Practical Nurses	34 (19.0)	24 (19.5)	4 (10.5)	17 (14.0)	
Certified Nursing Assistants	129 (72.1)	93 (75.6)	29 (76.3)	98 (81.0)	
**Time commitment**					4
Full-time	86 (48.3)	59 (48.4)	14 (37.8)	54 (45.0)	
Part-time	62 (34.8)	57 (46.7)	19 (51.4)	60 (50.0)	
Mixed	5 (2.8)	0	0	0	
Casual	25 (14.0)	6 (4.9)	4 (10.8)	6 (5.0)	
**Shift type**					10
Days	57 (33.0)	54 (44.3)	14 (36.8)	39 (33.1)	
Evenings	65 (37.6)	40 (32.8)	9 (23.7)	49 (41.5)	
Nights	30 (17.3)	10 (8.2)	9 (23.7)	22 (18.6)	
Mixed	21 (12.1)	18 (14.7)	6 (15.8)	8 (6.8)	
**Years worked in long-term care homes**					2
<1 year	36 (20.2)	7 (5.7)	1 (2.7)	15 (12.4)	
1–3 years	60 (33.7)	25 (20.3)	10 (27.0)	26 (21.5)	
4–10 years	72 (40.5)	53 (43.1)	14 (37.8)	53 (43.8)	
11–16 years	8 (4.5)	26 (21.1)	12 (32.4)	22 (18.2)	
17–25 years	2 (1.1)	11 (8.9)	0	5 (4.1)	
26+ years	0	1 (0.1)	0	0	
**Years worked in current unit**					4
<1 year	56 (31.6)	13 (10.6)	1 (2.7)	16 (13.3)	
1–3 years	68 (38.4)	26 (21.1)	11 (29.7)	29 (24.2)	
4–10 years	53 (29.9)	81 (65.9)	11 (29.7)	52 (43.3)	
11–16 years	0	3 (2.4)	14 (37.8)	23 (19.2)	
**Gender**					40
Female	153 (90.5)	108 (94.7)	29 (93.6)	97 (89.8)	
**Age**					43
<25 years	26 (15.4)	4 (3.6)	0	9 (8.3)	
25–34 years	80 (47.3)	9 (8.2)	5 (16.1)	14 (13.0)	
35–44 years	36 (21.3)	35 (31.8)	6 (19.4)	42 (38.9)	
45–54 years	25 (14.8)	44 (40.0)	10 (32.3)	25 (23.2)	
55–65 years	2 (1.2)	18 (16.4)	9 (29.0)	18 (16.7)	
>65 years	0	0	1 (3.2)	0	
I know the preferred habits for __ of my residents	4.0 (0.7)	4.1 (0.7)	3.6 (1.1)	3.6 (0.8)	13
I know __ of my residents’ favorite foods	3.3 (0.9)	3.8 (0.8)	3.3 (0.9)	3.4 (0.9)	17
I know ___ of my residents’ favorite music	3.0 (0.8)	3.2 (0.8)	2.9 (1.0)	3.0 (0.9)	16
I quickly help __ of my residents to the toilet when they request or need help	4.2 (0.8)	4.4 (0.7)	4.4 (0.9)	4.3 (0.7)	13
I know when __ of my residents need to use the toilet, even if they cannot speak	3.5 (0.8)	3.9 (0.9)	3.8 (0.8)	3.8 (0.8)	8
I can calm __ of my residents if they feel agitated or upset	3.8 (0.6)	3.9 (0.7)	3.5 (0.6)	3.6 (0.7)	7
I help __ of my residents stay connected to their families	3.8 (1.0)	3.9 (1.1)	3.7 (1.1)	3.6 (1.1)	17
I help __ of my residents stay connected to previous associations	2.8 (1.2)	2.8 (1.3)	2.6 (1.3)	2.2 (1.2)	40
I help __ of my residents keep family members as part of their life	3.8 (1.0)	3.9 (1.1)	4.1 (1.1)	3.8 (1.1)	20
I help __ of my residents spend time with people they like	3.9 (0.9)	4.0 (1.0)	3.9 (1.1)	3.7 (1.1)	17
I spend time talking or just being with __ of my residents	3.9 (0.9)	4.0 (0.8)	3.8 (1.1)	3.6 (0.9)	7
I look after the same residents from day to day	4.3 (0.8)	4.2 (0.7)	4.2 (1.0)	4.1 (0.9)	17
I am able to build fulfilling relationships with residents	4.3 (0.7)	4.2 (0.7)	4.1 (0.7)	4.0 (0.6)	12
I can learn from residents and their family members and incorporate this caring into my daily routine	4.2 (0.7)	4.2 (0.6)	4.0 (0.8)	4.0 (0.7)	19

**Table 4:** Rotated Component Pattern and Final Communality Estimates from Principal Component Analysis of the TM-PCC Survey.

Item	Description	1	2	3	h^2^
1	I know the preferred habits for __ of my residents	0.13	0.72	0.28	0.61
2	I know __ of my residents’ favorite foods	0.25	0.81	−0.01	0.71
3	I know ___ of my residents’ favorite music	0.33	0.71	0.00	0.62
4	I quickly help __ of my residents to the toilet when they request or need help	0.07	0.55	0.15	0.33
5	I help __ of my residents stay connected to their families	0.86	0.13	0.08	0.76
6	I help __ of my residents stay connected to previous associations	0.66	0.22	0.02	0.48
7	I help __ of my residents keep family members as part of their life	0.85	0.12	0.18	0.77
8	I help __ of my residents spend time with people they like	0.78	0.31	0.10	0.71
9	I look after the same residents from day to day	−0.04	0.11	0.63	0.41
10	I am able to build fulfilling relationships with residents	0.12	0.12	0.84	0.73
11	I can learn from residents and their family members and incorporate this caring into my daily routine	0.23	0.08	0.76	0.64

**Table 5:** Cronbach’s Alpha Coefficient and Descriptive Statistics.

Components	No. Items	Cronbach’s Alpha Coefficient	Mean	SD
Supporting Social Relationships	4	0.83	3.56	0.89
Familiarity with Residents’ Preferences	4	0.71	3.66	0.60
Meaningful Resident–Staff Relationships	3	0.62	4.17	0.56
